# Motor Imagery-Based Brain-Computer Interface Coupled to a Robotic Hand Orthosis Aimed for Neurorehabilitation of Stroke Patients

**DOI:** 10.1155/2018/1624637

**Published:** 2018-04-03

**Authors:** Jessica Cantillo-Negrete, Ruben I. Carino-Escobar, Paul Carrillo-Mora, David Elias-Vinas, Josefina Gutierrez-Martinez

**Affiliations:** ^1^Instituto Nacional de Rehabilitación, Division of Medical Engineering Research, 14389 Mexico City, Mexico; ^2^Instituto Nacional de Rehabilitación, Division of Neurosciences, 14389 Mexico City, Mexico; ^3^Centro de Investigación y de Estudios Avanzados del IPN, Section of Bioelectronics, 07360 Mexico City, Mexico

## Abstract

Motor imagery-based brain-computer interfaces (BCI) have shown potential for the rehabilitation of stroke patients; however, low performance has restricted their application in clinical environments. Therefore, this work presents the implementation of a BCI system, coupled to a robotic hand orthosis and driven by hand motor imagery of healthy subjects and the paralysed hand of stroke patients. A novel processing stage was designed using a bank of temporal filters, the common spatial pattern algorithm for feature extraction and particle swarm optimisation for feature selection. Offline tests were performed for testing the proposed processing stage, and results were compared with those computed with common spatial patterns. Afterwards, online tests with healthy subjects were performed in which the orthosis was activated by the system. Stroke patients' average performance was 74.1 ± 11%. For 4 out of 6 patients, the proposed method showed a statistically significant higher performance than the common spatial pattern method. Healthy subjects' average offline and online performances were of 76.2 ± 7.6% and 70 ± 6.7, respectively. For 3 out of 8 healthy subjects, the proposed method showed a statistically significant higher performance than the common spatial pattern method. System's performance showed that it has a potential to be used for hand rehabilitation of stroke patients.

## 1. Introduction

Stroke is the first cause of disability worldwide [1]. Loss of motor function, known as hemiparesis, is one of the most disabling consequences of stroke, which usually affects both upper and lower limbs from one side of the body. If stroke patients engage in therapy in the first 6 months after the initial symptoms appear, they have a 70% chance of regaining motor function in their affected hand [2, 3].

Assistive technologies such as robotic devices could increase the number of patients that receive therapy within this time. In addition, robotic devices have produced stroke rehabilitation outcomes at least as effective as those achieved with traditional therapies [4]. Brain-computer interfaces (BCI) are another type of assistive technology; these systems provide an artificial communication channel between the brain and an external device such as a robotic orthosis [5, 6]. BCIs based on motor imagery (MI) of affected limbs have shown great potential as a tool for brain plasticity enhancement [7, 8].

MI is a mental rehearsal of movements of a limb, for example, the hand or foot, without muscle activation [9–11]. MI elicits distinctive patterns in the electrical activity of the sensory-motor cortex, mainly in the frequency bands known as mu (8–13 Hz) and beta (14–30 Hz) [9, 12]. An MI-based BCI system is comprised of four stages; the first one is an electrical signal acquisition module such as electroencephalography (EEG). EEG is a noninvasive technique, has a good time resolution, and is easy to accept by patients. Preprocessing is the second stage of a BCI system; in this stage, signal artefacts such as eye movements, power line noise, and muscle activity are filtered from EEG recordings [13, 14]. The third stage is encompassed by feature extraction methods which preserve only significant information of the BCI user's intentions. Finally, the fourth stage is the classification phase, in which the extracted features are interpreted as the BCI user's intentions; linear discriminant analysis (LDA) is the most used classification technique reported in BCI publications [15, 16]. One of the most effective feature extraction methods is the common spatial pattern (CSP) algorithm, which computes a set of spatial filters that optimally differentiate two classes of MI. To achieve good classification accuracies using the CSP algorithm, the temporal filtering of the EEG signal must be performed on a specific frequency band. Usually, this band is selected in the mu and beta frequency range. Two other parameters that need to be set up are the time interval from which the features are going to be extracted and the subset of spatial filters involved in the feature extraction process [17].

The performance of CSP can be enhanced by selecting subject-specific parameters. Therefore, modifications to the original CSP method have been proposed to include this aspect. One of such modifications is known as filter bank common spatial patterns (FBCSP); this method performs an automatic frequency band selection for temporal filtering of the EEG [18]. FBCSP algorithm employs a filter bank that decomposes the EEG into different frequency bands. Each frequency band is spatially filtered using the CSP algorithm; afterwards, the extracted features for each band are selected with either the Mutual Information-based Best Individual Feature (MIBIF) or the Mutual Information-based Rough Set Reduction (MIRSR) algorithms. Classification is performed only with the selected features [18, 19]. Although, FBCSP performance was higher than CSP, statistically significant differences were not observed between both methods. A bank of filters and CSP are useful for MI; however; in order to increase classification performance other feature selection algorithms could be implemented. Feature selection is an optimisation problem; therefore, artificial intelligence techniques, such as particle swarm optimisation (PSO), could be applied for finding a solution for it.

PSO was originally proposed by Shi and Eberhart [20] and inspired by the social behaviour of bird flocks while searching for food. PSO performs a search in the space of the problem, with the aid of a population (called swarm) of individuals (called particles). Each particle executes a search based on its current position and velocity in the search space. In each iteration (called generations), the position and velocity of the particles are updated according to their best previous position (local search) and the best position of the swarm (global search). In terms of EEG properties, PSO can be applied to select which combination of extracted EEG features provides higher classification accuracies if used as inputs for a classifier. In each iteration (generation), several combinations of selected EEG features (particles) comprise possible inputs for a classifier. After all combinations have been used to train the classifier, and afterwards test it, classification accuracies for each combination (or other optimisation metrics) can be used to compare the fitness of each combination of EEG features. With this fitness information obtained for each combination, a new set of EEG feature combinations can be generated (a new generation of particles), which could contain a solution with higher classification accuracy than the ones tested in previous generations. This process is repeated until a stop criterion (like a number of generations or achieving a fitness value) is met. To the authors' knowledge, few reported studies describe the use of PSO as a feature selection algorithm for BCI systems. For example, Wei and Wei propose a frequency band selection using PSO and CSP algorithms; selection is based on the best classification accuracies achieved by the BCI. They evaluated their method using MI information from publicly available dataset IVa from BCI competition III. Classification performances were higher using the frequency bands selected by PSO than the ones computed with a broader frequency band [21]. Atyabi et al. proposed a PSO-based method to reduce the number of electrodes and the number of features used for MI classification. The authors evaluated their method with datasets IVa and IIIa from BCI Competition III [22]. Xu et al. evaluated a PSO-based algorithm for CSP frequency band and time selection using a database comprised of finger MI of 18 healthy subjects. They observed better offline performances with PSO than with a statistical approach for frequency and time band selection [23]. These works show that PSO can increase classification performance of MI while also decreasing the number of employed features; therefore, PSO could be a good feature selection method for BCI.

Recent studies have reported better motor rehabilitation outcomes using MI-based BCIs coupled to robotic assistive devices than the ones achieved with only robotic assistive devices [24, 25]. Some of the advantages of these combined systems are that they are noninvasive, are fully automated, and could increase brain plasticity. Some studies have evaluated the performances of these BCI systems with healthy subjects [6], as well as some proofs of concept [26, 27] and a randomised controlled trial [28] that have demonstrated positive rehabilitation outcomes for stroke patients. Even though BCI systems coupled to robotic assistive devices have shown promising outcomes for stroke rehabilitation, to date, none of such systems are used in clinical practice.

Reasons for this include the fact that most BCI systems are still under development in research centres and universities, are usually assessed offline, and have quite different performances in online tests. In addition, new processing stages designed for stroke rehabilitation BCI systems are not tested with EEG information of these patients. Therefore, tests must be performed to evaluate if an MI-based BCI is capable of classification of user's intentions and activation of external devices in online implementations, with a processing stage previously tested with stroke patients' data.

In this work, an MI-based BCI is implemented and tested; the system is aimed to be driven by hand MI. A novel signal processing stage comprised of a bank of filters, CSP for feature extraction, PSO for feature selection, and LDA for classification was designed. The proposed algorithm was evaluated offline with a sample of stroke patients' and healthy subjects' data and afterwards online tests were performed with the healthy subjects as users of the system. For online tests, MI was used to activate a robotic hand orthosis to evaluate the feasibility of the complete BCI system aimed for neurological rehabilitation of stroke patients.

## 2. Materials and Methods

### 2.1. Participants

The sample for this study comprised 8 healthy subjects (mean = 23.9 ± 1.3 years) and 6 patients diagnosed with stroke (mean = 55.8 ± 12 years). Both healthy subjects and stroke patients were required to have a normal performance in the subscales of digit detection and visual detection of the neuropsychological test NEUROPSI (this test has been validated for Spanish-speaking population) [29]. In addition, all subjects were naïve to BCI, with normal or corrected to normal vision, without any history of other neurological/psychiatric diseases and right-handed (at least 90% according to the Mexican adaptation of the Edinburgh handedness inventory [30]). In order to be considered for inclusion in the study, patients had to have a first stroke event of subcortical localisation, confirmed by a neurologist by means of neuroimaging studies (magnetic resonance or computed tomography) and total or partial paresis of one of their hands. Subcortical stroke patients were selected since their brain damage does not involve the brain cortex and, therefore, they were less likely to present significant cognition impairments. Before the EEG recordings were performed, the participants signed an informed consent approved by the Ethics and Research Committee of the National Institute of Rehabilitation in Mexico. Clinical and demographic data of the patients are shown in Table 1.

### 2.2. EEG Acquisition

**Figure 1:**
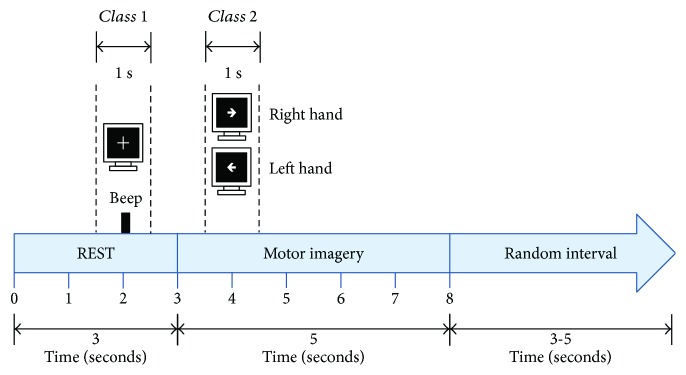
Illustration of the experimental paradigm. Dotted lines show the time windows extracted from EEG signals.

A g.USBamp biosignal amplifier from g.tec was used for EEG acquisition. EEG was acquired with 24 bits of resolution and sampling rate of 256 Hz. Active EEG electrodes were used for acquisition, with 11 electrodes placed over the scalp of the participants, in positions C3, C4, Cz, T3, T4, F3, F4, Fz, P3, P4, and Pz of the international 10–20 system. Ground placement was set in the AFz position, and the reference electrode was placed in the right earlobe. To verify that no real movements were elicited during MI, electromyography (EMG) was recorded from the *flexor digitorum superficialis* and *flexor digitorum profundus* muscles of both forearms. For the offline tests, each healthy subject participated in two sessions and performed in consecutive days with 120 trials recorded in total. To avoid exhaustion, stroke patients participated in four recording sessions which were performed in consecutive days, with 120 trials recorded in total. For the online tests, healthy subjects performed in consecutive days two additional sessions, with another 120 trials recorded in total. Subjects were instructed to sit in a comfortable armchair, with a computer monitor placed at 1.5 m in front of them. They were directed, by visual cues shown in the monitor, to perform either rest with their open eyes or MI from their paralysed hand (dominant hand in case of healthy subjects). EEG acquisition was performed using a similar strategy as the one followed by the Graz paradigm [31].  Figure 1 shows that the rest interval of the trials lasted 3 s and the MI interval lasted 5 s.

### 2.3. Offline Implementation and Validation of the Processing Stage

**Figure 2:**
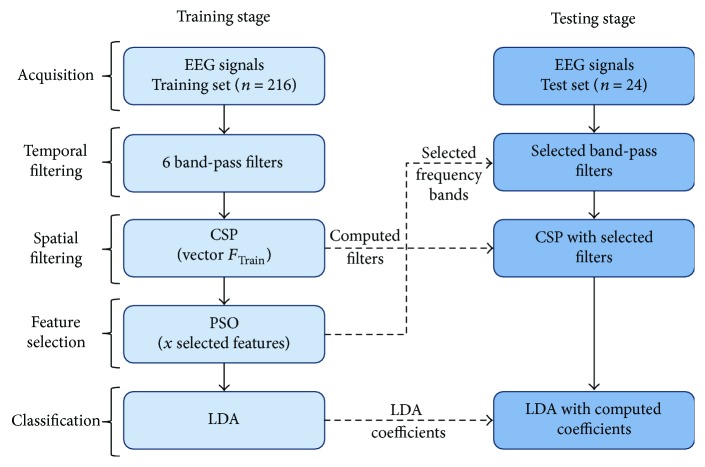
Block diagram of FBCSP + PSO implementation.

For offline implementation, a window of one-second length was extracted from 1.5 s to 2.5 s to obtain the rest information for each trial (REST). Another window of one-second length was extracted from the 3.5 to 4.5 s time interval of each trial, to obtain the MI information of the trials, as observed in Figure 1. These time windows were selected based on previous studies which show that differentiation between MI and REST classes is higher in these time intervals [32]. The FBCSP algorithm encompassed the processing stage of the BCI system, and PSO was used for feature selection (named FBCSP + PSO). A block diagram of the algorithm's implementation is shown in Figure 2.

The following is a detailed description of the algorithm's implementation:

(a) Temporal filtering: EEG data were filtered to obtain 6 frequency subbands, each 4 Hz broad and with 1 Hz of overlapping in order to avoid loss of information. Encompassing both alpha and beta frequency bands were as follows: 8–12 Hz, 12–16 Hz, 16–20 Hz, 20–24 Hz, 24–28 Hz, and 28–32 Hz. A 60 Hz band-stop filter was also applied to the EEG signals. All filters were FIR filters of the 30th order, selected for their linear phase features.
(b) Spatial filter: For the EEG data filtered in each subband, spatial filters were computed with the CSP algorithm. CSP performs a linear transformation on the EEG data, to obtain features whose variances are optimal for classification of two classes of MI, in a specific frequency band. Details of the CSP implementation can be found in the works of Blankertz et al. [17] and Ramoser et al. [33]. In this work, spatial filters were computed using the MATLAB command **W** = eig (*S*1, *S*1 + *S*2) as suggested in the abovementioned works. **W** is the matrix containing the spatial filters, and *S*1 and *S*2 are the covariance matrices of MI and REST computed from the EEG data of each filtered frequency subband. In the implementation of the original CSP, only the first and last *m* columns of the **W** matrix (*m* is generally 2) are used to generate the feature vector used for classification. With the goal of having a greater chance of finding the optimal subband for each patient, in this work, all possible features were extracted with CSP. The feature vector generated in this work for each trial *i* is comprised as follows:
  (1) $$\begin{array}{c} f_{i}=\left[f_{1,i},f_{2,i},f_{3,i},f_{4,i},f_{5,i},f_{6,i}\right]. \end{array}$$
Therefore, CSP features computed for the training set comprised for *nt* trials are
  (2) $$\begin{array}{c} F_{Train}=\left[f_{1};f_{2};f_{3};f_{4};\ldots ;f_{nt}\right],\;F_{Train}\in {\mathbb{R}}^{nt\times 66}. \end{array}$$
And the true class vector of the training set is
  (3) $$\begin{array}{c} y_{Train}=\left[y_{1};y;y_{3};y_{4};\ldots ;y_{nt}\right]. \end{array}$$
(c) Feature selection: PSO was used for selecting a subset of features from *F*_Train_, in order to decrease both the classification error and the number of selected features. Equation (4) describes PSO implementation.
  (4) $$\begin{array}{c} v_{i}^{n+1}=w\cdot v_{i}^{n}+c_{1}\cdot r_{1}\cdot \left({PBest}_{i}^{n}–x_{i}^{n}\right)+c_{2}\cdot r_{2}\cdot \left({GBest}_{g}^{n}–x_{i}^{n}\right),\\ x_{i}^{n+1}=x_{i}^{n}+v_{i}^{n+1}, \end{array}$$
  where *x*_*i*_^*n*^ and *v*_*i*_^*n*^ are the position and velocity of the *i*th particle of the *n*th generation. In this work, 50 particles and 50 generations were used. *w* is the inertial weight of PSO which linearly descends from 0 to 1 as generations of PSO are computed. *c*_1_ and *c*_2_ are positive constants set to 1. *r*_1_ and *r*_2_ have random values between 0 and 1, which coupled to *c*_1_ and *c*_2_ set the local and global search properties of PSO. PBest_*i*_^*n*^ is the best position reached by the *i*th particle in the *n*th generation. GBest_*g*_^*n*^ is the best position (*g*) reached by the entire swarm in the *n*th generation. The maximum position value that a particle could reach was 1 and the minimum was 0. Maximum speed of each particle was set to 1 and minimum speed to 0. In this work, the search space of PSO was 1 × *D*, where *D* equals 66 and was comprised of the 66 features that can be selected. Each computed solution with PSO is a subset of the selected features. Solution values are in the range from 0 to 1. If the value of an element of the solution was higher or equal to 0.5, then the corresponding feature was selected. The original CSP algorithm states that selected features must be paired, so in this work, complementary features of the selected ones were also included, in case they were not originally selected by PSO. Selected features from the training set were used for designing an LDA classifier. PSO fitness value was computed using
  (5) $$\begin{array}{c} value=\left(err\times 2\right)+\left(\frac{nselec}{66}\right), \end{array}$$
  where err is the computed classification error from the training set. nselec is the number of selected features. Variables err and nselec/66 have values ranging from 0 to 1. Both parameters err and nselec are summed so that PSO is able to perform a reduction of both classification error and the number of features used for classification. The err value was multiplied by 2 so that the optimization priority of PSO is the reduction of the classification error over the selection of a lower number of features. The stop criteria used for PSO was either achieving 0% of classification error or 50 generations.
(d) Classification: With the final selected features (*x*) and the training set, an LDA classifier was designed, which was later evaluated with the testing set. Features selected with PSO in the training stage were the same as the ones used for the testing stage of the classifiers. LDA performance was measured by computing the percentage of classification accuracy (%CA). In this offline stage, the necessary parameters for the online stage were computed. These parameters were the spatial filters for each frequency subband and the LDA coefficients.

A stratified cross-validation of 10 × 10-fold was used to avoid bias in the computation of %CA. Classifiers were tested using totally different datasets than the ones used for training. For each fold and repetition, the FBCSP + PSO algorithm was calculated. The 100 values of %CA obtained from this procedure were used to compute the average %CA for each participant.

The performance of the FBCSP + PSO was compared with that of the original CSP (on filtered data between 8 to 32 Hz) using the same training and test subsets. A preliminary version of the proposed algorithm was presented by Cantillo-Negrete et al. [34].

### 2.4. Robotic Hand Orthosis

Since rehabilitation of stroke patients with robotic assistive devices has advantages, a right-hand robotic orthosis was developed in previous works to couple it with the BCI [35, 36]. This orthosis comprised a 3D printed frame of polylactic acid (PLA). The orthosis linear actuators can provide passive flexion and extension movements to the fingers. A closed-loop system was used to sense the moment in which each finger reaches its maximum extension or flexion. The orthosis has a wireless Bluetooth communication with the processing stage of the BCI. The Bluetooth protocol was programmed in both MATLAB® and in a microcontroller attached to the electronic control circuit of the orthosis. The orthosis has four different actuators; however, for this study, all actuators were set to perform flexion and then extension of the hand fingers.

### 2.5. Online Implementation of the Designed BCI System

A graphical user interface (GUI) was programmed using MATLAB, comprised of user's/patient's data, a processing stage, a screen for visual cues presentation, and wireless Bluetooth configuration. Communication was established with the g.USBamp amplifier by means of an adaptor API (available from g.tec). The online processing stage was optimised to process windows of one-second length of the EEG signal. Healthy subjects' spatial filters and LDA coefficients computed for each selected frequency subband in the offline stage were programmed in the online BCI system. The BCI paradigm used for the online implementation was the same as the one used for the offline one, with the addition of the feedback provided by the robotic orthosis. For the online implementation, 6 windows of one-second length each were analysed. The first 3 windows comprised REST and the next 3 windows for MI. As soon as each window had elapsed, data recorded from them were processed in the GUI using the proposed FBCSP + PSO method and the LDA classifier. Afterwards, a classification output was generated which indicated if the time window was classified as REST or as right hand MI. Therefore, for each trial, the system performed 6 classification outputs. A total of 20 trials were recorded for each run, and 3 runs were performed for each session. A total of 2 sessions were performed in consecutive days recording 720 LDA outputs for the REST class and 720 for the MI class. Online %CA was computed per healthy subject by comparing the expected output with the real one.

**Figure 3:**
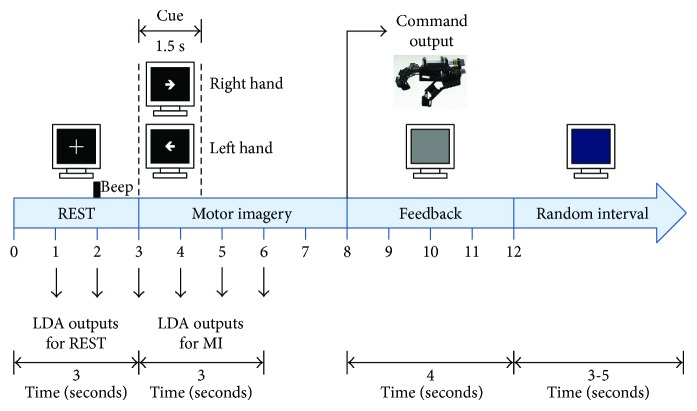
Timeline of the online BCI system, depicting a single trial.

The robotic hand orthosis was activated by a Bluetooth command sent from the computer, immediately after the MI time interval, only if 2 out of 3 motor imagery time windows were correctly detected by the processing stage. Within the orthosis activation time, the monitor displayed a grey background. The percentage of trials in which the orthosis was activated, regarded as percentage of correct trials (%CT), was computed for all the 120 trials per healthy subject. Activation of the orthosis was comprised of the opening and closing of the healthy subjects' fingers. After each run (20 trials), performance feedback was shown in the computer monitor by using faces with different degrees of smiling expressions. For example, if %CT was in the 100–90 interval, the most smiling face was shown to the subject, and if %CT was below 60%, the most serious face was shown. A depiction of the online processing stage is shown in Figure 3.

Averaged online processing time was computed for all the performed trials. This was the average time required by the preprocessing, FBCSP + PSO, and LDA stages to generate a classification output. A PC with a Core i5 processor of 2.53 GHz and 8 GB of RAM was used for running the GUI with the BCI processing stage.  S1 Video shows the complete BCI system in an online test.

### 2.6. Statistical Analysis

In order to assess the reliability of the BCI system, the practical level of chance was computed as explained by Müller-Putz et al. [37]. This practical level of chance is defined as the upper confidence interval of a random classifier's accsuracy. Practical level of chance was calculated with a binomial distribution using a significance level of 0.5, with 120 trials encompassing the data of each class. Equations (5) and (6) show the computation of the practical level of chance.

(6) $$\begin{array}{c} \tilde{p}=\frac{k+2}{n+4}, \end{array}$$

(7) $$\begin{array}{c} Practical\;level\text{ of }chance=\tilde{p}+\sqrt{\frac{\tilde{p}\left(1-\tilde{p}\right)}{n+4}\;}\left(1-\frac{\alpha }{2}\right), \end{array}$$

where $\tilde{p}$ is the probability of correct classification, *k* is the expected number of correctly classified trials, *n* is the number of trials, *z*_1−*α*/2_ is the 1 − *α*/2 quantile of the standard distribution, and *α* is the level of significance. The computed %CAs were compared with the practical level of chance to assess if BCI performance was significantly higher than chance [37].

A Lilliefors-corrected Kolmogorov-Smirnov test (*α* = 0.05) was used to test if stroke patients' and healthy subjects' %CAs for offline tests (obtained from 10 × 10-fold cross-validation) followed a Gaussian distribution. The tests showed that the offline %CAs computed with FBCSP + PSO, and CSP for both groups did not have a Gaussian distribution. Therefore, nonparametric Mann–Whitney *U* tests (*α* = 0.05) were used for comparing the offline %CAs computed with FBCSP + PSO and CSP. A Lilliefors-corrected Kolmogorov-Smirnov test (*α* = 0.05) showed that healthy subjects' offline and online averaged %CAs had a Gaussian distribution. Therefore, a paired *t*-test (*α* = 0.05) was used for comparing offline with online %CAs. Pearson's correlation and linear regression analyses were performed for measuring relationships between online %CA and %CT.

## 3. Results

### 3.1. Offline Analysis

**Figure 4:**
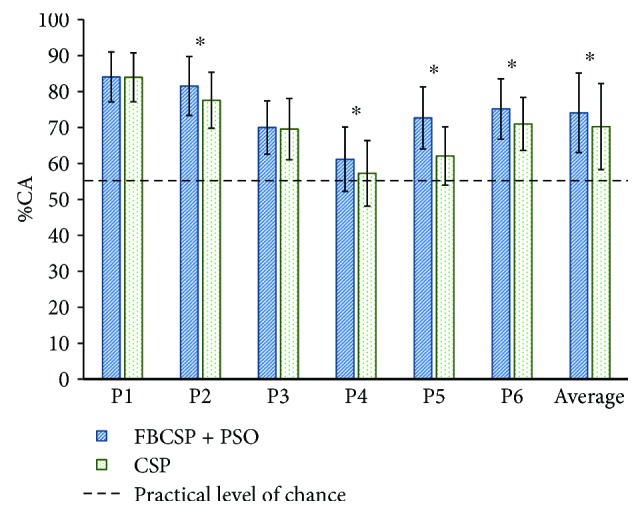
Offline classification accuracy percentages (%CAs) computed with data from 6 patients. The dotted line shows the practical level of chance (56.2%). The asterisk (^∗^) indicates that the %CA of FBCSP + PSO was statistically significantly higher (*p* < 0.05) than CSP.

Figure 4 shows the offline %CA computed with stroke patients' data with FBCSP + PSO and CSP. Results of the statistical analysis are also shown. The calculated practical level of chance for all experiments was 56.2%. It can be observed that for all patients %CAs were above the practical level of chance (*p* < 0.05) for both methods. For 4 out of 6 patient's data, %CA for the FBCSP + PSO algorithm was statistically significantly higher (*p* < 0.05) than the %CA for the CSP algorithm. For 2 patients, there were no statistically significant differences (*p* < 0.05) between the %CA for both methods. The averaged %CA for all stroke patients computed with FBCSP + PSO (74.1 ± 11%) was statistically significantly higher (*p* < 0.05) than the %CA obtained with CSP (70.2 ± 12%).

**Figure 5:**
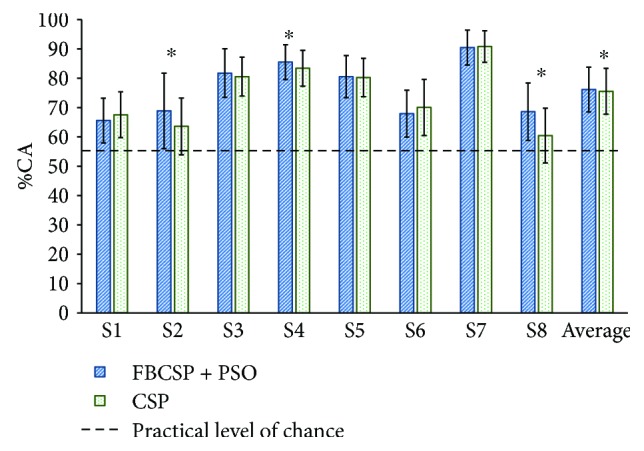
Offline classification accuracy percentages (%CAs) computed with data from 8 healthy subjects. The dotted line shows the practical level of chance (56.2%). The asterisk (^∗^) indicates that the %CA of FBCSP + PSO was statistically significantly higher (*p* < 0.05) than CSP.

Figure 5 shows the offline performance for healthy subjects' using the FBCSP + PSO and CSP algorithms. The %CA for all subjects were above the practical level of chance (*p* < 0.05) with both algorithms. For 3 out of 8 healthy subjects, FBCSP + PSO was statistically significantly higher (*p* < 0.05) than CSP. For the other subjects, no statistically significant differences (*p* < 0.05) were found between the %CA for both methods. Averaged %CA for the FBCSP + PSO method (76.2 ± 7.6%) was statistically significantly higher (*p* < 0.05) than the one obtained with CSP (75.5 ± 7.8%).

### 3.2. Online Analysis

**Figure 6:**
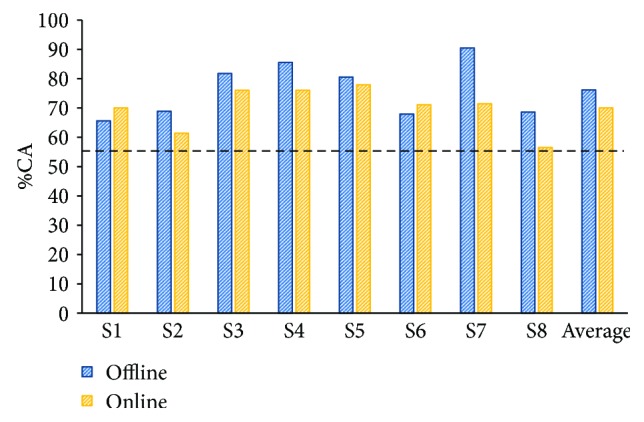
Online and offline classification accuracy percentages (%CAs) computed with data from 8 healthy subjects. The dotted line shows the practical level of chance (56.2%).

Figure 6 shows healthy subjects' online and offline performances. For all subjects, online %CA was above the practical level of chance (*p* < 0.05). Averaged offline %CA (76.2 ± 7.6%) was higher compared to online tests (70 ± 6.7%); however, there was no statistically significant difference (*p* > 0.05) between them.

**Figure 7:**
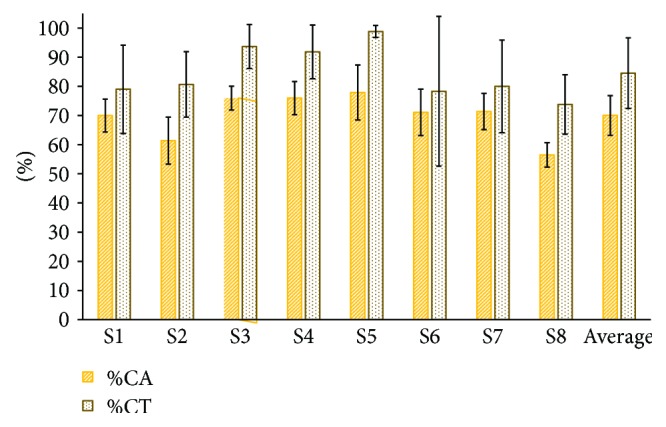
Online classification accuracy percentages (%CA) and correct trial percentages (%CT) from 8 healthy subjects.

Figure 7 shows subjects' online %CA (70 ± 6.7%) and %CT (84.5 ± 12.1%). Using Pearson's analysis, a 0.8 correlation between %CA and %CT was found. In addition, a linear regression analysis showed an *r* ^2^ value of 0.64. Averaged online processing time was of 0.04 ± 0.01 s.

## 4. Discussion

The proposed BCI system was tested with stroke patients' and healthy subjects' data. Offline performances computed with FBCSP + PSO and CSP were above the practical level of chance for all stroke patients and healthy subjects. However, for 4 out of 6 patients, FBCSP + PSO showed a statistically significant higher performance of their paralysed hand compared to CSP. In addition, 3 out of 8 healthy subjects' offline tests using FBCSP + PSO also showed statistically significant higher performances than CSP. Furthermore, for none of the stroke patients and healthy subjects, FBCSP + PSO performance was significantly lower than CSP. Ang et al. also performed an evaluation of their FBCSP using MIRSR for MI classification. Their algorithm was tested with a public database comprised of 9 healthy subjects and compared with the performance of CSP. However, FBCSP trained with MIRSR performances were not statistically significantly higher than CSP (with a 7 to 35 Hz frequency band) [19], unlike FBCSP + PSO in which the performance was statistically significantly higher than CSP. Therefore, FBCSP + PSO could be a better option for an MI BCI processing stage than CSP since it showed significantly higher performances for both stroke patients and healthy subjects.

The heuristic nature of PSO implies that its performance will not be limited by statistical features of the search space, since the method does not need to compute inverse matrices or other computations which often present restrictions, especially for high dimensional search spaces. Offline performances of the BCI system show that PSO implementation for feature selection of FBCSP allows this method to have better performances than CSP. This performance is achieved by using a multiobjective optimisation for the PSO algorithm by setting a higher importance to the LDA's classification than to the number of selected features in the fitness function.

The system's average processing time (0.04 ± 0.01 s) was lower than the time window used for EEG acquisition (1 s), which makes possible the online implementation of the system.

Stroke patients' offline performances (74.1 ± 11%) were similar to the ones reported by Ang et al. with a sample of 46 stroke patients, which achieved an average offline performance of 74% using 27 EEG channels and an FBCSP with MIBIF algorithm. However, in the present work, only 11 EEG channels were recorded. In addition, stroke patients' offline performances were higher than online performances reported using other state-of-the-art BCI designs. For example, Morone et al. performed an acquisition of 61 EEG channels from 8 stroke patients. The goal of the study was to assess if the recruited patients could perform an online grasping of a virtual hand. They reported an average %CA of 57 ± 24% [26]. Performances computed with the proposed BCI were also higher than the ones reported by Zhang et al. They recruited 8 stroke patients for the evaluation of a BCI coupled to a functional electrical stimulator. They processed 19 EEG channels with a modified CSP algorithm for feature extraction and support vector machines for classification. The average performance of their BCI system was 66% [38].

Healthy subjects' average online performance (70 ± 6.7%) was higher than the one reported in a study with a similar feedback, using a hand exoskeleton by Witkowski et al. [39]. In the study, a %CA of 67.4% was reported (63.59 ± 10.8 of sensitivity and 71.3 ± 11.02 of specificity), using 5 EEG channels, in a sample of 12 healthy subjects. In another study with a hand exoskeleton feedback reported by Tang et al., healthy subjects' online performance was 84.29 ± 2.11%. However, only 4 subjects with good MI ability were recruited and 24 EEG electrodes were used [40]. Healthy subjects' online performances using the proposed BCI system were not significantly lower than the offline ones. Therefore, the proposed FBCSP + PSO processing stage should be able to handle the increased signal artefacts present in an online acquisition.

Offline and online tests allow us to suggest that stroke patients' online performance with the proposed BCI is likely to be around 70% or at least higher than the practical level of chance.

Online %CT was positively correlated with the %CA of the system, which implies that the feedback shown to users reflected their ability to voluntary elicit hand MI. This is important since showing a correct feedback to patients motivates them to keep a successful MI strategy or to seek different approaches if feedbacks indicate low performances.

This study showed that the proposed FBCSP + PSO processing stage and robotic orthosis feedback are suitable for a BCI aimed for neurorehabilitation. However, tests involving patients using the system are still required to evaluate its neuroplasticity-enhancement capabilities. The participants of these future tests should include patients with cortical stroke located in the dominant and nondominant hemisphere. The observed performance differences show that FBCSP + PSO could be a better option than CSP for feature extraction in an MI-based BCI. However, online acquisition data from a higher sample of patients participating in a randomized controlled trial are still necessary to completely describe the potential of the proposed BCI system as a neurorehabilitation tool for stroke patients. Another study limitation is that 2 sessions were performed per participant, and a higher number could provide information on performance variations across time. Therefore, the next step in the system's assessment should be to define a therapy schedule, which should include the lessons learned from this study which are to use FBCSP + PSO as processing stage, %CT for patient's feedback, 3 runs of 20 trials each per day, and a somatosensory feedback using a robotic hand orthosis.

## 5. Conclusions

This work presents a BCI system evaluation using a processing stage comprised of FBCSP + PSO combined with LDA and feedback provided by a robotic orthosis. PSO as a feature selection algorithm for FBCSP allows reducing the problem's dimensionality and achieving better classification performances, compared to those obtained if only the original CSP is used.

The present study shows that with the proposed BCI design patients are likely to be able to control a hand robotic orthosis using motor imagery of their paralysed hand. Therefore, the next developing stage of the system will be to perform a randomised controlled study involving direct EEG acquisition from patients. The BCI system designed in this study combines the advantages of a robotic device and motor imagery, which have separately produced good results for stroke patients' rehabilitation. Therefore, if the proposed BCI system design is introduced into the clinical practice it would provide medical facilities with a tool that could aid stroke patient's functional recovery.

## Figures and Tables

**Table 1:** Patients' clinical and demographic data.

Patient	Gender	Age	Hemiparesis	Evolution	Injury location
1	Male	50	Right	7 months	Posterior limb of left the internal capsule
2	Female	57	Right	36 months	Left pulvinar nucleus of the thalamus extending to the left internal capsule and ipsilateral lateral ventricle
3	Male	58	Left	2 months	Right basal ganglia with involvement of the posterior limb of ipsilateral internal capsule
4	Female	79	Left	1 month	Posterior limb of the right internal capsule
5	Male	46	Left	3 months	Lenticular nucleus, internal capsule, and right corona radiata
6	Male	45	Left	3 months	Right side of the brainstem

## Supplementary Materials

Supplementary Materials S1 Video shows a user of the proposed BCI system while performing right motor imagery. The robotic hand orthosis, computer screen with visual cues, and the graphical user interface can also be observed. In each trial, if the classifier stage of the BCI detected subject's motor imagery, passive hand finger flexion and extension was provided by the orthosis. After all trials of one session had been presented to the user, its BCI performance was shown on the computer screen.
